# Outcomes in orthopedic device infections due to *Streptococcus agalactiae*: a retrospective cohort study

**DOI:** 10.1186/s12879-024-09175-6

**Published:** 2024-04-22

**Authors:** Ava Diarra, Benoit Gachet, Eric Beltrand, Julien Dartus, Caroline Loiez, Elise Fiaux, Pierre Patoz, Olivier Robineau, Eric Senneville

**Affiliations:** 1 Department of Infectious Diseases, Hôpital Gustave Dron, 135 rue du Président Coty, F-59200 Tourcoing, France; 2 ULR 2694 - Évaluation des technologies de santé et des pratiques médicales, Univ. Lille, F-59000 Lille, France; 3 Department of Orthopedic Surgery, Hôpital Gustave Dron, Tourcoing, France; 4 Department of Orthopedic Surgery, CHRU de Lille, Lille, France; 5 Department of Bacteriology, CHRU de Lille, Tourcoing, France; 6 Department of Infectious Diseases, Centre Hospitalo-Universitaire de Rouen, Rouen, France; 7 Department of Bacteriology, Gustave Dron Hospital, Lille University, Lille, France

**Keywords:** *Streptococcus agalactiae*, Group B streptococci, Joint infection, Prosthetic

## Abstract

**Background:**

Group B streptococci (S*treptococcus agalactiae*) (GBS) is a rare cause of prosthetic joint infection (PJI) occurring in patients with comorbidities and seems to be associated with a poor outcome. Depiction of GBS PJI is scarce in the literature.

**Methods:**

A retrospective survey in 2 referral centers for bone joint infections was done Patients with a history of PJI associated with GBS between 2014 and 2019 were included. A descriptive analysis of treatment failure was done. Risk factors of treatment failure were assessed.

**Results:**

We included 61 patients. Among them, 41 had monomicrobial (67%) infections. The median duration of follow-up was 2 years (interquartile range 2.35) Hypertension, obesity, and diabetes mellitus were the most reported comorbidities (49%, 50%, and 36% respectively). Death was observed in 6 individuals (10%) during the initial management. The rate of success was 63% (26/41). Removal of the material was not associated with remission (*p* = 0.5). We did not find a specific antibiotic regimen associated with a better outcome.

**Conclusion:**

The results show that *S. agalactiae* PJIs are associated with high rates of comorbidities and a high treatment failure rate with no optimal treatment so far.

## Introduction

Group B streptococci (GBS) or *Streptococcus agalactiae* is a well-known cause of infection among neonates and pregnant women. Although GBS joint infections are rare, representing 4–12% of periprosthetic joint infections (PJIs) mostly hematogenous (12–39%) rather than exogenous (1–6%) [1, 2]. They were the most frequent *Streptococcus* species (34%) in a multicenter study of PJI [3]. In the literature, the data on GBS joint infection involving a material is scarce and focused on joint prostheses. A previous case series showed that a majority of them had an underlying disease and a high reinfection rate (60%) [4]. A retrospective study on 163 patients revealed that infections predominantly occurred in patients aged > 50 years with comorbidities. The place of surgery depends on the duration of symptoms and the implantation date of prosthesis [5]. The antibiotic treatment recommended is penicillin G or ceftriaxone IV or oral amoxicillin [6]. To our knowledge, there are no recommendations that addressed the question of the benefit of the rifampicin-levofloxacin combination in the treatment of GBS-related PJIs. In the present study, we aimed to describe the management of GBS implant-related orthopedic infections and patient’s outcome.

## Materials and methods

The patients were treated for bone and joint infections at Tourcoing General Hospital and Lille University Hospital from 2014 to 2019, both hospitals serving as the North-West French National Referent Center for complex bone and joint infections (CRIOAC Lille-Tourcoing). Patients were included if *S. agalactiae* was isolated from per-operative samples and/or joint aspiration performed before the surgery. The patients underwent surgical intervention consisting of debridement, antibiotics, and implant retention (DAIR) of one or two-stage reimplantation, implant removal, bone resection, arthrodesis, and amputation. During surgical procedures, at least 3 tissue samples were taken in different areas suspected of being infected, using a separate sterile instrument for each sample. Each sample was cultured for five days at 35 °C in Columbia agar with blood 5% and chocolate agar with polyvitex; and for 14 days in aerobic and anaerobic bottles incubated in the Virtuo blood culture system (BioMérieux, Marcy l’Etoile, France) for Lille Hospital patients, Rosenow broth for Tourcoing Hospital patients (Biorad, Marnes la Coquette, France). Strains were identified using MALDI-TOF spectrometry mass (Bruker Daltonics, Wissembourg, France for Lille strains and Vitek MS BioMérieux, Marcy l’Etoile, France for Tourcoing strains) with a minimum score requirement of 2. The antibiotic susceptibility profile of all pathogens identified from intraoperative samples was assessed either by the Vitek 2 cards (BioMérieux, Marcy l’Etoile, France) or by the agar diffusion technique using the procedure and interpretation criteria proposed by the Comité de l’Antibiogramme de la Société Française de Microbiologie (CA-SFM-EUCAST) (http://www.sfm-microbiologie.org).

### Definition

PJIs were defined according to the 2012 IDSA guidelines [6] and classified as early (≤ 3 months), delayed (between 3 months and 2 years), and late (more than 2 years). Acute postoperative infection occurred by definition ≤ 4 weeks [7]. Patients’ data were collected until the latest news was available in the medical file. Remission was defined as the absence of signs of infection (e.g. fever, edema, erythema, non-healing wound, fistula) at the initial site. Relapse and recurrence were defined as the occurrence of the PJI involving the same bacteria respectively, within and more than 6 months after empirical antimicrobial treatment (EAT). Superinfection was defined as the occurrence of an infection at the same location but due to pathogen(s) distinct from the initial one(s). Failure was defined by recurrence, relapse, superinfection, and any other situations than remission, especially the need for any further surgical procedure related to the infection (i.e. a second DAIR in case of initial DAIR option, implant removal, or amputation), the need for a suppressive long-term antibiotic treatment or death related to the initial infection. The patient’s outcome was determined when the course of evolution was unfavorable or at the time of last known consultation. Outcome events corresponded to failure diagnosis and last check-point in case of remission after a duration of at least 1 year of infection-free survival.

### Data management and statistics

We used the electronic database of the Lille-Tourcoing CRIOAC to collect the patients’ characteristics, microbiology, surgical intervention, and antibiotic therapy. We compared remission and failure (relapse, reinfection, superinfection, and infection-related death) groups around comorbidities, type of infection (early, delayed, or late) antibiotic therapy, and surgery. Categorical variables are expressed in terms of frequency and percentage. Quantitative variables are expressed as means ± standard deviation (SD) or medians (med) and interquartile range (IQR). Categorical data were compared with the chi-square test or Fisher’s exact test. Quantitative variables were compared with a t-test. We conducted the analyses with R 4.0.3.

## Results

### Characteristics of patients and clinical presentations of the infections

A total of 61 patients with implant-related orthopedic infections due to mono or polymicrobial GBS were identified in our database. The demographic characteristics and comorbidities of the patients as well as the pathogens involved in the joint infections are shown in Table 1. Our study reveals that obesity (26/52, 50%) hypertension (21/43, 49%), and diabetes mellitus (21/59, 36%) were the most frequent comorbidities before the infection. Two-thirds of the patients (41/59, 67%) had a monomicrobial infection. Polymicrobial infections with GBS were predominantly involving *Staphylococcus* spp. and gram-negative bacilli (GNB). Fifteen patients out of 45 (33%) had a previous infection of the peri-prosthetic tissue.

**Table 1:** Characteristics of 61 patients with orthopedic implant infection due to *S. agalactiae*

Patients’ characteristics	*n* = 61
Sex M/F [n (%)]	22 (36) / 39 (64)
Age (years) [med (IQR)] out of 61 patients	67 (16)
ASA score [med (IQR)] out of 50 patients	2 (1)
BMI kg/m² [mean (SD)] out of 52 patients	32 (8)
**Underlying disease**	
Diabetes mellitus [n (%)]	21/59 (36)
Cirrhosis [n (%)]	1/59 (2)
Cancer [n (%)]	7/59 (12)
Chronic renal failure [n (%)]	6/59 (10)
GFR < 30 mL/min [n (%)]	4/37 (11)
Immunosuppressant therapy [n (%)]	8/57 (14)
Chronic cardiac disease [n (%)]	9/47 (19)
Chronic obstructive pulmonary disease [n (%)]	2/47 (4)
Inflammatory rheumatism [n (%)]	3/59 (5)
Alcohol abuse	3/43 (7)
Hypertension	21/43 (49)
Obesity ^a^	26/52 (50)
**Microbiological characteristics**	
***S. agalactiae*mono-infection** [n (%)]	39/59 (66)
**Polymicrobial infection** [n (%)]	20/59 (34)
*Staphylococcus aureus* [n (%)] out of 20 polymicrobial infections	11 (55)
*Staphylococcus epidermidis* [n (%)] out of 20 polymicrobial infections	7 (35)
*Enterococcus* spp. [n (%)] out of 20 polymicrobial infections	2 (10)
*Cutibacterium acnes* [n (%)] out of 20 polymicrobial infections	1 (5)
Gram negative bacilli [n (%)] out of 20 polymicrobial infections	6 (30)
**Clinical manifestation**	
Fever [n (%)]	29/51 (57)
Local inflammation [n (%)]	35/43 (81)
Fistula [n (%)]	15/55 (27)
**Inflammatory biomarkers**	
Leukocytes (G/L) [med (IQR)] out of 37 patients	8 (10)
Polymorphonuclear (G/L) [med (IQR)] out of 13 patients	4 (2)
CRP (mg/L) [med (IQR)] out of 33 patients	133 (186)
**Radiological evidence**	
Osteolysis [n (%)]	3/21 (14)
Implant loosening [n (%)]	3/20 (15)
Luxation [n (%)]	0/20 (0)
**Per-operative purulent exudate**	20/34 (59)
**Prosthetic device**	
Prosthesis [n (%)]	47/61 (77)
Hip arthroplasty [n (%)]	27/61 (44)
Knee arthroplasty [n (%)]	23/61 (38)
Ankle arthroplasty [n (%)]	1/61 2(2)
Osteosynthesis [n (%)] Internal fixation device (medullary nailing device) [n (%)]	9/61 (15) 3/61 (5)
Arthrodesis [n (%)]	1/61 (2)
Duration between implantation and infection (weeks)	
≤ 3 months (early infection) [n (%)]	4/52 (8)
> 3 months [n (%)] ≤ 2 years (delayed infection) [n (%)] > 2 years (late infection) [n (%)]	48/52 (92) 23/52 (44) 25/52 (48)

ASA: American Society of Anesthesiologists; BMI: Body Mass Index, GFR: Glomerular Filtration Rate, IQR: interquartile range, Med: median, SD: standard deviation

Obesity was defined as Body Mass Index > 30 kg/m2

IQR: interquartile range, Med: median, SD: standard deviation

Details on the clinical, radiological, and per-operative presentation are presented in Table 1. Most of our patients’ cohort had signs of inflammation at the onset of the infection. On the contrary, biological and radiological signs were sporadic among patients, except for the C-Reactive-Protein (CRP) elevation. The vast majority (48/52, 92%) of the infections occurred at the delayed (23/52, 44%) or late (25/52, 48%) stage.

### Infection’s management

The details on the type of surgery were available for 50 of them (Table 2). DAIR was the most favored therapeutic option (19/50, 37%) in our patient cohort, followed by one-stage exchange arthroplasty (18/50, 36%). Of note, of all the DAIR performed only 2 (11% of the total DAIR) concerned early infections and 1 acute post operative infection. The proportion of hematogenous infection was not mentioned. The adapted following pathogen’s antibiogram oral antibiotic therapy was detailed for 53 patients. Among them, a majority of patients received rifampin combined therapy including 31 (58%) with levofloxacin. Thirty-seven patients with monomicrobial infection had a known adapted *per os* treatment. Among them, 27 patients received a rifampin combination and 22 rifampin-levofloxacin association (59%). When focusing of patients with 1 year of follow-up (41), 27 (51%) and 21 (65%) received rifampin combination and rifampin-levofloxacin association. All strains with available antibiograms (55/61, 90%) were susceptible to culture-guided antibiotic treatment.

**Table 2:** Medical and surgical therapy of 61 orthopedic implant infections due to *S. agalactiae* and outcomes of 54

Management	[n (%)]
**Surgery**	
DAIR [n (%)] Performed on early infection (out of total DAIR) Performed on delayed or late infection	19/50 (38) 2/18 (11) 16/18 (89)
One-stage exchange arthroplasty [n (%)] Performed on early infection (out of total One-stage exchange arthroplasty) Performed on delayed or late infection	18/50 (36) 2/18 (11) 16/18 (89)
Two-stage exchange arthroplasty [n (%)]	10/50 (20)
Bone resection [n (%)]	1/50 (2)
Arthrodesis [n (%)]	1/50 (2)
Amputation [n (%)]	1/50 (2)
**Antibiotic therapy**	
Post-operative *intravenous* (IV) therapy - Cefotaxime [n (%)] - Cefepime [n (%)] - Daptomycin [n (%)] - Ceftobiprole [n (%)]	13/34 (38) 5/34 (15) 12/34 (35) 5/34 (15)
IV duration (days) [med (IQR)] out of 33 patients	6 (7)
Post-operative *per os* therapy - Rifampin combined with other antibiotics than Levofloxacin^a^ - Levofloxacin combined with other antibiotics than Rifampin or alone - Rifampin-levofloxacin combination	6/53 (11) 12/53 (23) 31/53 (58)
Oral treatment duration (weeks) [med (IQR)] out of 54 patients	12 (8)
**Outcomes with at least 1 year follow-up**	*n* = 41
Remission [n (%)]	26 (63)
Relapse [n (%)]	1 (2)
Reinfection [n (%)]	5 (12)
Superinfection [n (%)]	3 (7)
Suppressive antibiotic therapy [n (%)] - Amoxicillin (n) - Clindamycin (n) - Doxycycline (n) - Dalbavancin-ertapenem combination (n)	8/41 (20) 4 1 1 2

DAIR: Debridement, Antibiotics, Implant Retention

^a^ combination when described included amoxicillin, minocycline

### Outcomes

After a median duration of follow-up was 2 years (IQR 2.35, minimum 1 day maximum 6 years), 35 (57%) patients were in remission. The treatment of the infection failed in 12 cases (22%) including 3 relapses, 6 reinfections, and 3 superinfections. Among those failures 2 cases ended in an amputation and 3 cases in an arthrodesis. The median duration between infection and failure was 8 months (IQR 4.5). Three superinfections were described including 1 with methicillin-resistant *Staphylococcus epidermidis*. Five of the 6 deaths in our cohort were related to infections and associated with septic shocks. Of note, all the deaths occurred in less than 2 months (median duration between death and infection of 26 days). One was linked to gastro-duodenal hemorrhage. Eight patients (19%) received antibiotic suppressive therapy, among them, 3 achieved remission during the follow-up.

When focusing on patients with at least 1 year follow-up (41), no distribution of comorbidities was significantly different between success and failure of treatment. There was no difference between early vs. delayed or late infections in terms of success. Patients in remission were not significantly more treated with rifampin-levofloxacin (14/26 remission vs. 7/12 failure; *p* = 0.7) regardless of the surgical protocol. The rifampin-based combination was not associated with a better outcome when compared to other regimens more used in patients in remission (9/12 vs.18/26 failure; *p* > 0.9 0.2). Implant removal was not associated with a better outcome (13/19 remission vs. 6/12 failure; *p* = 0.5). When analysis focused only on monomicrobial infections, no significant differences were shown (rifampin-levofloxacin 11/20remission vs. 5/8 failure; *p* > 0.9; rifampin-based combination 15/20 remission vs. 6/8failure *p* > 0.9; implant removal 8/13remissions vs. 3/6 failure *p* > 0.9).

## Discussion

Consistently to the literature, patients with GBS infection in our cohort had multiple comorbidities including obesity, hypertension, and diabetes [8–10]. Our study revealed that patients with a prior infection of the prosthesis had the worst outcome. We confirmed that GBS PJIs have poor outcomes as already suggested for Streptococcal PJIs[ 3]. We did not find a significant proportion of patients in remission with the rifampin regimen, whatever the surgical treatment. No standardized antibiotic therapy has been recommended for GBS infection on materials so far. However, similarly to staphylococcal PJIs, we argue that optimizing the oral switch therapy with antibiotics with high oral bio-availability, good bone distribution, and potential anti-biofilm activity would be of interest as recently reported [11]. Our study has several limits; firstly, the retrospective collection of data and the scarcity of some collected data might be sources of bias and impairment of the analysis, notably the proportion of hematogenous infection. Secondly, the heterogeneity of patients in terms of monomicrobial and polymicrobial infection prevents a focused analysis of GBS. One additional limit is that we did not use sonication or dithiothreitol methods which are more sensitive than intraoperative tissue cultures to detect polymicrobial infection. Larger studies on a bigger scale need to evaluate, through a randomized trial, the potential of the rifampin-fluoroquinolone combination in these patients and more generally in PJIs not related to Staphylococcal strains.

## Data Availability

The datasets used and/or analyzed during the current study are available from the corresponding author on reasonable request.
